# The rise and fall of long-latency *Plasmodium vivax*

**DOI:** 10.1093/trstmh/trz002

**Published:** 2019-02-27

**Authors:** N J White

**Affiliations:** Mahidol Oxford Research Unit, Faculty of Tropical Medicine, Mahidol University, Bangkok, Thailand; Centre for Tropical Medicine and Global Health, Nuffield Department of Medicine, University of Oxford, Oxford, UK

**Keywords:** long latency, malaria, *Plasmodium vivax*, relapse

## Abstract

Until World War II the only clinical phenotype of *Plasmodium vivax* generally recognised in medicine was one associated with either a long (8–9 months) incubation period or a similarly long interval between initial illness and the first relapse. Long-latency *P. vivax* ‘strains’ were the first in which relapse, drug resistance and pre-erythrocytic development were described. They were the infections in which primaquine radical cure dosing was developed. A long-latency ‘strain’ was the first to be fully sequenced. Although long-latency *P. vivax* is still present in some parts of Asia, North Africa and the Americas, in recent years it has been largely forgotten.

## The discovery of long latency in vivax malaria

In 1922, at the Wythenshaw mental hospital, near Manchester, England, Professor Warrington Yorke and Dr John William Scott MacFie began a remarkable series of therapeutic evaluations. They started by infecting local anopheline mosquitoes with quartan malaria parasites (*Plasmodium malariae*) from a patient from West Africa, and on 21 July they allowed these mosquitoes to bite two patients suffering from general paralysis of the insane.^1^ Two weeks later the first patient became ill and a few days after that malaria parasites were detected in their peripheral blood. On 8 September they infected their first patient with *Plasmodium vivax*, which became their preferred infection after the one patient they infected with *Plasmodium falciparum* developed disease of ‘fulminating character’ and died.^1^ All their patients, like tens of thousands of others in psychiatric hospitals across the world, were suffering from neurosyphilis, and Yorke and MacFie were hoping to cure them. At the time, neurosyphilis was a uniformly progressive and ultimately lethal condition, but there was recent hope. This had come initially from Paul Ehrlich’s discovery of his ‘magic bullet’, the arsenical ‘Salvarsan’ (arsphenamine—which also had activity against some ‘strains’ of *P. vivax* malaria), but more recently from the studies of the Viennese neuropsychiatrist Julius Wagner-Jauregg on ‘malariotherapy’, for which he would win the Nobel Prize in 1927.^2^ He reported that recurrent high fevers could alleviate or cure neurosyphilis, so Yorke and many others were eager to emulate these extraordinary claims.^3^ Wagner-Jauregg used malaria-infected blood to infect his patients, and initially so did Yorke and MacFie, but when the relatives of the patients with neurosyphilis in the Wythenshaw hospital objected, they turned to mosquito infections. They infected the local malaria vectors *Anopheles maculipennis* and *Anopheles bifurcatus* (now *Anopheles claviger*). Malaria parasites appeared in the blood between 13 and 21 days after their patients were bitten by the infected mosquitoes, and the patients became febrile. After a short course of quinine (3 d), relapses occurred after intervals of 3–6 weeks in 4 of the 31 survivors (initially only severely ill patients were infected and thus there was a high early mortality from neurosyphilis). A total of 41 patients were infected with *P. vivax* in this way. There was some improvement in several of the patients’ neuropsychiatric conditions, so Yorke and MacFie duly published their results.^1^ Over the next year, however, something unexpected happened. In more than half the mosquito-infected patients the *P. vivax* infection recurred, while none of the 61 patients who had been infected by blood had a recurrence of malaria. The recurrences after 2 months (we may now call them late relapses) occurred a median of 8 (range 3–100) months after the initial infections.^4^

These were not the first artificial malaria infections of humans in England. In 1900 Patrick Manson wanted to allay widespread scepticism over the recent claim by his protégé Ronald Ross that dapple winged (anopheline) mosquitoes could transmit malaria. In a well-publicized demonstration, Manson tested the ‘mosquito theory’ by allowing malaria-infected anopheline mosquitos to feed on his son Patrick Thurburn Manson (then a medical student at Guy’s hospital) and another volunteer in London. These malaria-infected *Anopheles maculipennis* mosquitoes had been kindly sent (in the Embassy bag) from Rome via Brindisi by his fellow malariologists Grassi, Bignami and Bastianelli, who had fed them originally on one of their patients with vivax malaria in the Ospedale di Santo Spirito. This Italian *P. vivax* ‘strain’ caused malaria illness in 2 weeks as hoped, providing confirmation of the Ross findings in India.^5^ Some 9 months later the younger Manson, who had since moved to Aberdeen, began to feel ill with malaise and abdominal discomfort. Two days later he experienced a sudden onset of chills (the ‘paroxysm’) and a pathologist duly confirmed by microscopy of his blood that he was suffering again from vivax malaria.^6^ These findings (of long-latency relapse) were consistent with other detailed case reports and observations from Europe by Celli in Italy and, in particular, Korteweg in The Netherlands,^7^ who proposed that malaria in the early summer months was acquired in the late summer or early autumn of the previous year. Korteweg’s detailed prospective individual observations in the village of Wormeveer, allied with Swellengrebel’s detailed entomological studies, showed clearly that the surge of vivax malaria that occurred in the early summer months preceded the abundance of vector mosquitoes.^8^ They concluded that the early summer malaria could only have been acquired by mosquito bites in the previous year and therefore that these infections had an incubation period of 8–9 months.

**Figure 1.**
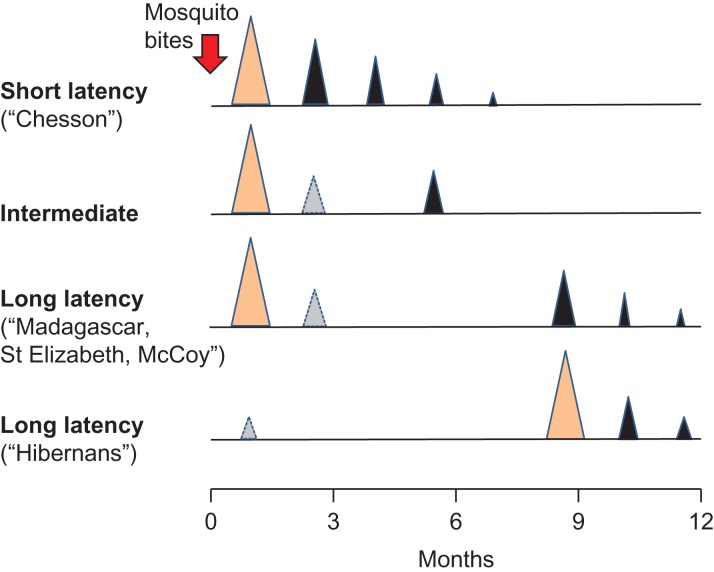
General temporal pattern of initial illness (light brown) and relapses (black) with different *P. vivax* phenotypes mainly characterized from volunteer/malariotherapy studies in which infections were usually with 5–10 infected mosquitoes (red arrow). Intervals are those associated with slowly eliminated antimalarial treatments (chloroquine, mepacrine, piperaquine). The light grey triangles depict relapses occurring in a minority of cases. Early relapse in long-latency *P. vivax* was more likely with heavy sporozoite inocula, although some strains never relapsed early (<2 months) in volunteer/malariotherapy studies.

This concurred with increasing malariotherapy experience in both Europe and the USA and detailed characterization of the St Elizabeth and McCoy strains in the US and the Madagascar strain in Europe (Table 1).^9–11^ The latter is probably the single most studied malaria parasite in history. Beginning on 25 May 1925, under the supervision of the UK’s leading malariologist, Sidney Price James, at ‘C Hospital’ (later the Horton Malaria Laboratory, Epsom, UK), anopheline mosquitoes, which had earlier fed on a seaman of Indian origin whose last port of call was Madagascar, were allowed to bite two women with neurosyphilis.^12^ So began a remarkable sequence of infections spanning more than 20 y (1739 patients and 24 361 mosquitoes were infected). Although this parasite lineage was called the ‘Madagascar strain’, as Garnham has pointed out, it may well not have originated on the island of Madagascar.^13^ When transmitted by mosquitoes, this ‘strain’ caused an initial illness, sometimes an early relapse, but more commonly a late relapse about 8–9 months after the original infection (Figure 1). With increasing human passage the ‘Madagascar strain’ apparently became more virulent.^14,15^

**Table 1.** Mean (standard deviation or range) relapse intervals from last antimalarial treatment dose to patency (days) recorded for drug-treated infections with various strains of *P. vivax* in human volunteers: modified from Schmidt^25^

*P. vivax* strain	Antimalarial	P–R_1_	R_1_–R_2_	R_2_–R_3_	R_3_–R_4_
St. Elizabeth (USA)	Quinine	272 (42)	11 (2)	17 (7)	36 (16–56)
	Chloroquine	256 (36)	66 (13)	53	
Korea	Chloroquine	293 (17)^a^	68		
Nicaragua	Quinine	108 (33)	20 (2)	21 (20–21)	20 (19–21)
Panama	Quinine	185 (37)	23 (23–23)		
Salvador^b^	Quinine	217 (20)	24		
Chesson (Papua New Guinea)	Quinine	9 (2)		22 (26)	
Chesson	Chloroquine	67 (13)	71 (11)	114 (78)	96 (33)
Chesson	Amodiaquine	57 (18)	67 (21)	75 (24)	97 (35)
Chesson	Proguanil	24 (2)	27 (2)	32 (7)	49 (28–70)

^a^From Arnold et al.^31^

^b^This was the first *P. vivax* to be sequenced (*Sal 1*)^43,44^ and is now the reference genome.

P: primary illness; R: relapse.

In those early days, well before discovery of the malaria parasites’ exoerythrocytic development, there was uncertainty as to the cause of malaria recurrence and also some confusion over terminology (relapses were defined by the interval from the primary infection, not biology). It was also unclear why some infections had a long latency between primary illness and recurrence and others apparently had a long incubation period. Swellengrebel and his colleagues (including his wife) resolved the uncertainty over the incubation period by allowing themselves to be bitten by single mosquitoes infected with a local Netherlands ‘strain’ of *P. vivax*. They remained well for approximately 8 months before all falling ill with vivax malaria.^8^ The temporal pattern of illness was determined by both geographic origin and the dose of sporozoites received. The more sporozoites that were administered, the greater was the likelihood of early illness and the greater the chance of relapse. This was, and it still is, an important confounder in epidemiological assessments.

## The discovery of radical cure

In the two decades before World War II, detailed chemotherapy studies were conducted by the Indian Medical Service, mainly in Kasauli, Himachal Pradesh, Northern India (which lies at 2000 m, an altitude above the transmission zone). This series of clinical trials, led mainly by John Alexander Sinton,^16^ together with earlier studies in Lahore, suggested a different pattern of recurrent vivax malaria to that documented in Europe and the USA. The infections ‘relapsed’ frequently at short intervals. Most of the patients studied were soldiers who had already endured multiple relapses (in hindsight, these infections were probably a mixture of short- and long-latency ‘strains’). Sinton and his colleagues found that the newly discovered synthetic 8-aminoquinoline antimalarial drug pamaquine (plasmochin, plasmoquine), when given together with adequate doses of quinine, could prevent most of these relapses (thereby providing a ‘radical cure’).^17,18^ Subsequent deployment of a radical curative malaria treatment regimen throughout the British army in India led to a dramatic reduction in malaria illnesses.^19^ Sinton also advocated strongly for standard malaria treatment regimens and standard methods of assessment.^16,19^ But this was not accepted by the Malaria Commission of the League of Nations. The committee, comprising mainly malaria experts from Europe, were strongly influenced by their experiences with detailed follow-up of neurosyphilis patients in Europe and reports from North America. They did not accept initially that relapse could be effectively and safely prevented by pamaquine.^20^ They also did not accept that there could or should be standard malaria chemotherapeutic regimens—they thought treatment required tailoring to the individual.^20^ The committee believed that all *P. vivax* had the propensity for late relapse to a greater or lesser extent. The more tropical ‘strains’ had an incubation period of a little less than 2 weeks, sometimes relapsed early, but then usually did not relapse again for more than 6 months (Table 1). Further north, where summer mosquito breeding seasons are short, the long-latency parasites gave way to *P. vivax* with a long incubation period (later called *P. vivax hibernans*).

## The discovery of frequent-relapse *P. vivax*

The belief that all *P. vivax* could have long-latency relapses persisted until World War II, in which, just as in World War I, soldiers on both sides were badly affected by malaria. The allied research effort was centred in the USA, where there was a desperate search for new drugs to prevent recurrent malaria. The US research programme, under the Office of Scientific Research and Development, was led by James Shannon, who would later become the head of the entire National Institutes of Health. New drugs were evaluated in malaria-infected neurosyphilis patients and later, when larger numbers were required, in prison volunteers.^21^ Multiple relapses of vivax malaria were a particular problem for the US forces, who, unlike the British, Indian and Australasian forces, did not recommend radical curative treatment with pamaquine. The US authorities may have been ‘spooked’ by the serious outbreak in May 1943 of blackwater fever in imported labourers working in the Panama Canal zone who had all been given pamaquine.^22^ Safer alternatives were needed.

Shannon regarded relapsing vivax malaria as the main threat to the military in the Pacific theatre. He was frustrated by the need to wait up to 9 months for the pharmacodynamic read-out (i.e., relapse) in evaluating potential radical curative treatments with the native US long-latency *P. vivax* infections. So he sought help from the Medical Research Unit (Australian Imperial Force), Cairns, Australia, where Neil Hamilton-Fairley and his Australian army colleagues were studying the treatment of vivax malaria in soldiers who had acquired it fighting on the island of New Guinea.^23^ This vivax malaria in Australian soldiers relapsed at 3-week intervals after quinine and approximately 6-week intervals after treatment with the slowly eliminated quinacrine (mepacrine, atebrine). Fairley wrote: ‘The strain or strains of P. *vivax* encountered in New Guinea and New Britain differed from the strains encountered in Australian troops in the Middle East and those previously worked-with experimentally in U.K. and U.S.A. inasmuch as the relapses appear within a few weeks of primary fever or the cessation of suppressive drug therapy; though febrile attacks are readily controlled by anti-malarial drugs the subsequent relapse rate is unduly high’.^23^

Shannon was sceptical initially that *P. vivax* malaria could behave in this way, but he recognized the potential of these ‘unusual’ strains for drug testing. Fairley suggested he look for frequent relapsing *P. vivax* in returned GIs, and so Sargent Chesson, hospitalized in the Harmon General Hospital (Longview, TX, USA) with malaria that he had acquired in New Guinea, was asked if he would kindly donate a blood sample containing the malaria parasites that would later bear his name.^24^ There followed intense studies of the ‘Chesson strain’ in prison volunteers in the Stateville Penitentiary (Jolliet, IL, USA) and also of an animal model, *Plasmodium cynomolgi* in rhesus monkeys, which seemed very similar to this frequent-relapse Chesson strain of *P. vivax* in humans.^24–26^ With the same pattern as the infections studied by Sinton in India, the Chesson strain of *P. vivax* relapsed at intervals of 3–6 weeks depending on the elimination profiles of the antimalarial drugs used in treatment (Figure 1). But there was no evidence of long latency.

## The discovery of primaquine

The war was soon over and so the military imperative for antimalarial drug discovery decreased. Penicillin was gradually supplanting malariotherapy for the treatment of neurosyphilis. There was an epidemic of long-latency *P. vivax* in The Netherlands,^27^ but otherwise it was the tropical frequent-relapse Chesson type of *P. vivax* that held centre stage in the research programmes as they gradually wound down.^28^ Development work on new 8-aminoquinolines had given pentaquine and isopentaquine, which had slightly better therapeutic ratios than their predecessor pamaquine. These compounds were overtaken when, in comparative studies conducted in the *P. cynomolgi* model, and later in prison volunteers, the simpler molecule SN 13272 (now called primaquine) appeared significantly more efficacious and also less toxic than pamaquine.^29^

Then came the Korean War. This necessitated a resurgence of interest in long-latency *P. vivax* for, while there was no falciparum malaria in the Koreas, there was plenty of long-latency/long incubation period *P. vivax*. The United Nations’ soldiers became heavily infected. A surge of malaria infections occurred 9 months after their intense exposure. Then, as had been observed previously in Europe and North America, once the first long-latency relapses occurred, subsequent relapses followed at short intervals (i.e. a similar pattern to the tropical Chesson strains)^30,31^ (Figure 1). Primaquine was rushed into service for radical cure at an adult dose of 15 mg/day for 14 days, a dose that continues to be recommended to this day.^32,33^

After the Korean War, malaria research declined again as the World Health Organization’s global malaria eradication initiative began. Most of the research on vivax malaria focussed on the Chesson strain, although detailed malariotherapy studies in the USSR provided the most accurate characterization of the different long-latency phenotypes^34–36^ (Table 2). The Soviets characterized strains that had a short incubation period, strains that had long incubation periods and strains that could exhibit both phenotypes. Uniquely, they were also able to document a second period of long latency in some of the patients studied. The proportion of *P. vivax* infections caused by long-latency strains increased in some areas (e.g. Azerbaijan, Tajikistan) after the intense control efforts associated with the global eradication plan in the 1950s and 1960s and the resurgence of malaria in the following decades.^37^

**Table 2.** Relapse patterns characterized in the USSR (from Tiburskaja et al.^36^)

Parasite group	Name of strain	Where and when isolated	Climatic zone	Length of incubation period (days)	
				Short	Long
Strains invariably producing short incubation	Volgograd	Volgograd 1945	Temperate	11–24	
	Nahicevan	Nahicevan, Azerbaijan, 1937	Subtropical	12–19	
	Vietnam	North Vietnam, 1954	Subequatorial	14–25	
Strains invariably producing long incubation	Naro-Fominsk	Moscow Oblast, 1946	Temperate		250–401
	Kolomna	Moscow Oblast, 1937	Temperate		273–337
	Hlebnikovo	Moscow Oblast, 1948	Temperate		195–430
Strains producing both types of incubation with short predominating	Moscow	Moscow Oblast, 1953	Temperate	9–20	216–308
	Leninabad	Leninabad, Tajikistan 1950	Subtropical	10–24	257–355
Strains producing both types of incubation with long predominating	Korea	North Korea, 1953	Temperate	17–22	274–390
	Rjazan	Rjazan, 1945	Temperate	10–37	282–403

**Figure 2.**
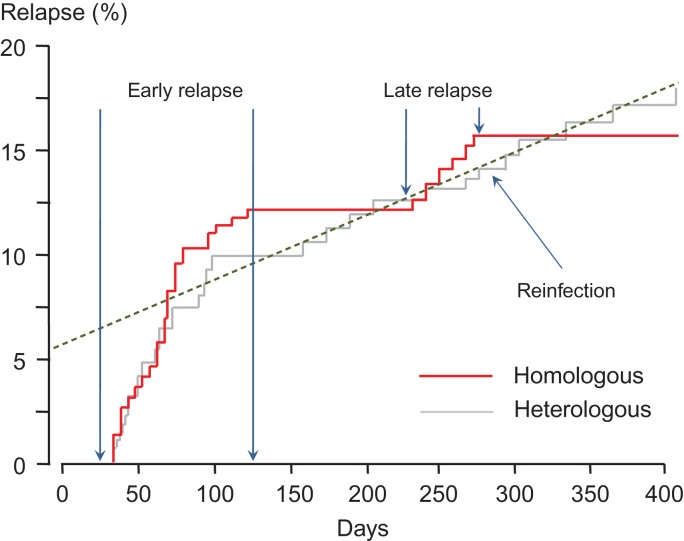
Relapse pattern following acute vivax malaria in Kolkata from Kim et al.^41^ The majority of relapses occurred within 100 d of the acute infection. Approximately half of these early recurrences were with parasites that were genetically related to the index infection, suggesting they arose from the same sporozoite inoculum (red line). The other half were genetically unrelated, suggesting activation of previously acquired hypnozoites or reinfection (grey line). There was a steady rate of reinfection throughout the year (dashed line). Beginning 8 months after the index infections, there were six temporally grouped genetically homologous relapses indicating the long-latency *P. vivax* phenotype. This suggests co-circulation of frequent-relapse (‘Chesson type’) and long-latency *P. vivax* parasites.

Documentation of different relapse patterns also continued in India, where *P. vivax* parasites with the ‘Madagascar’ long-latency phenotype appear to coexist with Chesson types and perhaps a third phenotype with intermediate characteristics and a shorter latency^38^ (Figures 1 and 2). But by the beginning of the 1990s, the long-latency phenotypes had been all but forgotten outside Russia, India and the Koreas. With a resurgence of global interest in malaria came belated research on *P. vivax*, but this has concentrated almost exclusively on parasites with short-interval relapses.

## The current situation

The number of people infected with the long-latency *P. vivax* parasites has declined markedly as malaria has been eliminated from Europe, North America, Russia, the Middle East, large areas of North Africa, Central Asia and parts of the Indian subcontinent. But it has not gone away. Long-latency *P. vivax* remains hidden in plain sight in the few remaining infections in Central America, admixed in South America, and in Central Asia, northern India and North Korea.^39,40^ It may also persist in North Africa (in Mauritania and Mali). It is easy to miss. Early relapse may occur with the subtropical strains of long-latency *P. vivax*, and is the norm for second and subsequent relapses (i.e. the patient may be presenting with a relapse rather than the primary infection as the apparent incident illness, so any subsequent relapses will have the same pattern as the tropical Chesson type of infection). As the first long-latency relapse is isogenic (i.e. identical or a sibling) compared with the initial infection, parasite genotyping in carefully followed patients can readily identify this phenotype (Figure 2).^41^ Discovering the genetic basis for the control of latency may prove difficult despite the rapidly increasing number of fully sequenced parasites (which are nearly all frequent relapse isolates, in contrast to the original *Sal 1*). Obviously if a marker is identified, it would facilitate therapeutic assessments. There is some evidence that the radical curative efficacy of primaquine is greater in long-latency infections, but this could also reflect geographic and epidemiological differences independent of latency (Table 3).

**Table 3.** Long latency *P. vivax*: outstanding questions

1. What is the current geographic distribution of long-latency *P. vivax*?
2. What is the molecular basis for latency?
3. What is the nature of the biological clock that results in an interval of >8 months between blood stage infections?
4. How do long-latency hypnozoites avoid death from hepatocyte apoptosis?
5. Is there an intermediate-latency (duration: 3–6 months) phenotype?
6. Is the radical curative efficacy of 8-aminoquinolines greater in long-latency compared with short-latency strains of *P. vivax*?
7. Are the anopheline vectors similar for long-latency and short-latency strains of *P. vivax*?

## Conclusions

The long-latency strains of *P. vivax* have played a critical role in our understanding of vivax malaria. It was in long-latency vivax malaria that relapse was first characterized properly, drug susceptibility and intrinsic resistance were first documented, the pre-erythrocytic stage of development of human malaria was discovered^42^, primaquine was first field tested, and the widely used dose of 0.25 mg/kg/d for 14 d was established. The first *P. vivax* chosen to be sequenced^43^ was a long-latency strain (*Sal 1*) from El Salvador.^44^*P. vivax* hypnozoites were demonstrated first in liver biopsies from two chimpanzees, one infected with the Chesson strain and the other with a long-latency North Korean strain.^45^ Despite all this, long-latency *P. vivax* has dropped off the radar in the past 30 y. A review entitled ‘Key gaps in the knowledge of *Plasmodium vivax*’ did not even mention long-latency or long incubation period *P. vivax*.^46^ Mapping the current geographic distribution of long-latency *P. vivax* will be important for both control and elimination planning and will also inform the durations of follow-up required in therapeutic assessments.
